# Structure-activity relationship of immunostimulatory effects of phthalates

**DOI:** 10.1186/1471-2172-9-61

**Published:** 2008-10-31

**Authors:** Søren T Larsen, Gunnar D Nielsen

**Affiliations:** 1 National Research Centre for the Working Environment, Lersø Parkallé 105, DK-2100 Copenhagen, Denmark

## Abstract

**Background:**

Some chemicals, including some phthalate plasticizers, have been shown to have an adjuvant effect in mice. However, an adjuvant effect, defined as an inherent ability to stimulate the humoral immune response, was only observed after exposure to a limited number of the phthalates. An adjuvant effect may be due to the structure or physicochemical characteristics of the molecule. The scope of this study was to investigate which molecular characteristics that determine the observed adjuvant effect of the most widely used phthalate plasticizer, the di-(2-ethylhexyl) phthalate (DEHP), which is documented as having a strong adjuvant effect. To do so, a series of nine lipophilic compounds with structural and physicochemical relations to DEHP were investigated.

**Results:**

Adjuvant effect of phthalates and related compounds were restricted to the IgG1 antibody formation. No effect was seen on IgE. It appears that lipophilicity plays a crucial role, but lipophilicity does not *per se *cause an adjuvant effect. In addition to lipophilicity, a phthalate must also possess specific stereochemical characteristics in order for it to have adjuvant effect.

**Conclusion:**

The adjuvant effect of phthalates are highly influenced by both stereochemical and physico-chemical properties. This knowledge may be used in the rational development of plasticizers without adjuvant effect as well as in the design of new immunological adjuvants.

## Background

**Figure 1:**
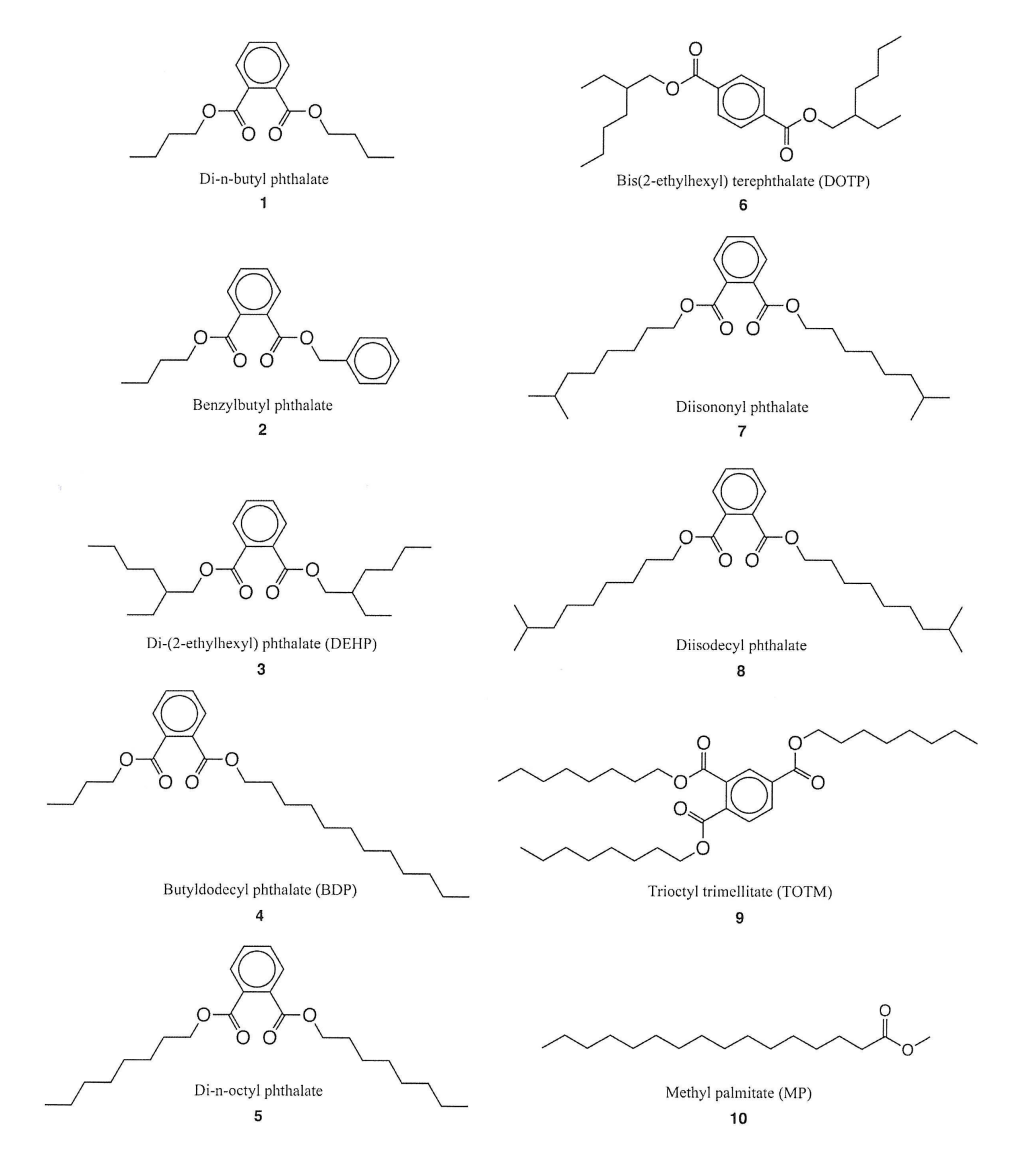
2-d structures of the studied compounds

Exposure to immunomodulatory environmental pollutants can promote development of allergy as reviewed by Nielsen et al. [1]. However, immunomodulation is also used intentionally to promote the efficacy of vaccines [2]. Phthalates, which are added for example to poly vinyl chloride (PVC) plastic polymers in order to increase the flexibility of the product, constitute a group of xenobiotics that have been in focus regarding their effect on the immune system. Phthalates are not themselves immunogenic [3], but phthalate plasticizers and metabolites hereof have been shown to enhance the effect of immunogens, which means they have an adjuvant effect [4-7]. Also, it has been suggested that phthalates play a role in the elicitation phase of allergy [8,9]. However, recent long-term animal studies have not confirmed an allergy (IgE) promoting effect of di-(2-ethylhexyl) phthalate (DEHP) or its main metabolites, mono-2-ethylhexyl phthalate (MEHP) [6,7]. Phthalate plasticizers with closely related structures showed marked variability in inducing production of specific IgG1 to a simultaneously administered allergen in mice [5,10,11]. Whether this variability to exert an adjuvant effect is due to lipophilicity, length of the ester chains in the phthalate or due to stereochemical characteristics is not clear. The aim of this study is twofold; a) to identify factors that contribute to the adjuvant effect of phthalate plasticizers with the view to enabling the design of compounds without adjuvant effect, and b) to allow development of compounds with maximum IgG1 promoting effects as they may be useful as lead compounds for vaccine adjuvants. The compounds included in this study are shown in Figure 1.

## Methods

### Animals

Inbred BALB/cJ female mice, 6–7 weeks old, were purchased from Bomholtgård Breeding and Research Centre, Denmark. The mice were randomly divided into groups of 10 to 12 and were housed in environmentally enriched polypropylene cages (425 × 266 × 150 mm) with pinewood sawdust bedding (Lignocel S8, Brogaarden, Denmark). The mice were kept in pathogen limited conditions and were allowed to acclimatize two weeks before immunization. At the time of immunization, the mean weight of the animals was 18 g with a SD of 1.6 g. The photoperiod was from 7 a.m. to 7 p.m., and the temperature and relative humidity in the animal room were 21 ± 2°C and 50 ± 5%, respectively. The cages were sanitized twice weekly. Mice were maintained on an ovalbumin (OVA) free diet (Altromin no. 1324, Christian Petersen, Denmark) and tap water was available *ad libitum*. Treatment of the animals followed procedures approved by The Animal Experiment Inspectorate, Denmark.

### Chemicals

Polyethylene glycol 400 (PEG, CAS 25322-68-3, Ph. Eur.) was purchased from Merck, Germany. Di-(2-ethylhexyl) phthalate (DEHP, CAS 117-81-7, purity ≥ 97%) was from Fluka, Buchs, Germany. Methyl hexadecanoate (MP, CAS 112-39-0, purity ≥ 99%) was from ABCR, Karlsruhe, Germany. Tri-(2-ethylhexyl) trimellitate (TOTM, CAS: 3319-31-1, purity ≥ 99%) and bis-(2-ethylhexyl) terephthalate (DOTP, CAS 6422-86-2, purity ≥ 96%) were both from Aldrich Chemical Company, Milwaykee, USA).

Butyl dodecyl phthalate is not commercially available but was synthesized at our laboratory as follows: 600 mg (1 molequivalent) of mono-n-butyl phthalate (CAS 131-70-4, purity>95%) synthesized at our laboratory according to [4] was dissolved in 10 mL acetone. To this mixture was added 223.8 mg (0.6 molequivalent) potassium carbonate. Furthermore 759 mg (= 0.95 molequivalent) of dodecyliodide (CAS 4292-19-7, purity ≥ 98% from Fluka, Buchs, Germany) was dissolved in 15 mL acetone and added to the monobutyl phthalate solution over a period of approximately 25 minutes (dripping from a burette) under continuous stirring.

As the synthesis included light sensitive chemicals, the reaction was carried out in the dark. The mixture was stirred for 24 hours to let the reaction complete. The mixture was filtered to remove excess K_2_CO_3 _and rotary evaporated. The residue was extracted with diethylether and the combined, yellow-colored ether phase was rotary evaporated leaving a pale yellow oil. The oil was dissolved in ether and purified by shaking it with saturated NaHCO_3_. The ether phase was dried with Na_2_SO_4_, filtered and vacuum evaporated. The final yield was 350 μL. The identity of the butyldodecyl phthalate was confirmed by proton nuclear magnetic resonance (^1^HNMR) spectroscopy. The purity of the product was more than 95% as determined by gas chromatography – mass spectroscopy (GC-MS) analysis. Impurities included dodecyl iodide and dibutyl phthalate.

### Immunization procedure

Mice were sensitized intraperitoneally (i.p.) by a standard low-dose protocol with the model allergen hen egg OVA (grade V from Sigma, St. Louis) alone or together with one of the test substances, which are shown in Figure 1. The OVA control mice were given 1 μg OVA in 50 μL solvent (vehicle), composed of sterile PEG 400, ethanol 99.9% and sterile water in a ratio of 494:5:1. As DEHP has previously been shown to have an adjuvant effect [10], it was used as a positive adjuvant control and reference substance. Mice in the test groups were given 1 μg OVA together with one of the test substances, using the same solvent as for the OVA and DEHP control groups. The respective concentrations of the test substances were 20 μg/mL, 200 μg/mL and 2000 μg/mL. As PEG 400 may have a mild immuno-suppressive effect [12], no more than 50 μL was administered to each animal leading to respective doses of test substance of 1, 10 and 100 μg per animal. After primary immunization, the animals were given one or two booster injections i.p. with 0.1 μg OVA in 100 μL 0.9% saline without test compound. The first booster injection was given 14 days after primary immunization, the second booster seven days later.

Blood was collected 14 days after the respective booster injections, i.e. day 28 and 35. Before collection of blood by heart puncture, the mice were anaesthetized with Hypnorm^® ^(fentanyl citrate 0.315 mg/ml and fluanisone 10 mg/mL from Janssen Pharma) and Dormicum^® ^(midazolam 5 mg/mL from Roche) each diluted 1:1 in sterile water and then mixed. A volume of 125 μL per mouse was injected s.c. in the neck region. The blood samples were centrifuged at 2500 × g for 10 min and the serum was stored at -80°C until analyzed.

### Determination of OVA-specific antibodies in sera

Levels of OVA-specific IgE, IgG1 and IgG2a antibodies were assessed as previously described [11].

### Statistical analysis and evaluation of adjuvant effect

For compounds that were tested at different times, new OVA control groups were included. This was done to normalize for possible time-dependent variations in the OVA control groups and differences in the protocols (cf. below). Antibody levels in test groups were compared to the corresponding OVA control group. The adjuvant effects of the tested substances were assessed by the induction of OVA-specific IgE, IgG1 and IgG2a.

The OVA control group and the three exposure groups (1, 10 and 100 μg) were first evaluated by means of the Kruskal-Wallis test. If significant differences were obtained, the exposure groups were then pair-wise compared to the OVA control group by means of Mann Whitney's U-test.

Minitab Statistical Software, Release 13.1 Xtra (Minitab Inc.), was used for the Kruskal-Wallis and the Mann-Whitney's U-tests. P-values less than 0.05 were considered statistically significant.

The adjuvant effect was expressed as the fold increase in the antibody production compared to the production in the corresponding OVA control group that received the same number of boosters (Table 1). The number of fold increases in antibody response is called the adjuvant factor throughout this article. To increase the amount of data used for the structure-activity relationship, data from previous studies [5,11] were included. These compounds (dibutyl phthalate, benzyl butyl phthalates, di-n-octyl phthalate, di-iso-nonyl phthalate and di-iso-decyl phthalate, cf. Table 1) were tested at slightly different conditions. With regard to the previously determined data, less time elapsed between booster injections in the immunization protocol as well as between the last booster injection until the collection of serum cf. [5]. In addition, the site of injection was subcutaneous in the neck region and not i.p. injection. The change of injection site was done to avoid skin irritation induced by the PEG-based vehicle. The immunization protocol was changed, as experiments performed in our laboratory showed that extension of the time periods lead to a protocol that was more robust and superior at revealing an adjuvant effect [13]. DEHP as well as Al(OH)_3 _were tested in both sensitization protocols, and it was found that the new protocol gave rise to a 6-fold higher IgG1 adjuvant factor with both compounds and thus can be used as a conversion factor. Thus, to enable comparison between our previously reported data and the data obtained in this study, the previous adjuvant factors were multiplied by a factor of 6.

**Table 1:** Adjuvant effect of test compounds based on the IgG1 levels.

Compound	Adjuvant factor^a ^(dose of test compound)	
	1 booster	2 boosters
1: Di-n-butyl phthalate	ND^b^	24 (10 μg)^c^
2: Benzyl butyl phthalate	1^d^	1^d^
3: Di-(2-ethylhexyl) phthalate (DEHP)		13 (10 μg)
	33 (100 μg)	61 (100 μg)
4: Butyldodecyl phthalate (BDP)		20 (10 μg)
	14 (100 μg)	68 (100 μg)
5: Di-n-octyl phthalate	ND	61 (100 μg)^c^
6: Bis-(2-ethylhexyl) terephthalate (DOTP)	1	4 (100 μg)
7: Diisononyl phthalate	ND	42 (both 10 and 100 μg)^c^
8: Diisodecyl phthalate	ND	1^c^
9: Trioctyl trimellitate (TOTM)	1	1
10: Methylpalmitate (MP)	1	1

a) Adjuvant factor taken as the ratio between the IgG1 level in the test group and the OVA control group.

b) ND: Not defined, as the median IgG1 value in the OVA control group was zero.

c) Data are calculated from reference 5. Cf. 'Material and Method' for details.

d) Data are calculated from reference 11. Cf. 'Material and Method' for details.

As all test compounds showed maximal adjuvant effect at the highest dose (cf. *Results*), this dose was used for the description of structure-activity relationships.

## Results

### Systemic effect

A decrease in body weight may be used as an indicator of general toxicity [14]. The body weights of the animals were therefore determined before and 24 hours after primary immunization. No dose-dependent change in body weight was observed in any of the test groups when these were compared to the OVA control groups. This indicates that no general toxic effects occurred after a single s.c. injection with up to about 5 mg/kg of the studied compounds.

### IgG1 responses

For DEHP an adjuvant factor of 33 (i.e. a 33-fold increase in the antibody level compared to the OVA control group) was observed in mice given 100 μg DEHP and boosted once. After two boosters this adjuvant factor increased to 61. Administration of 10 μg DEHP did not give rise to an adjuvant effect after one booster, but when boosted twice, the DEHP group had a level of IgG1 that was 13 times that of the OVA control group (cf. Table 1).

Comparable levels were seen for BDP, where an adjuvant factor of 14 was observed after 100 μg BDP and one booster. After two boosters, adjuvant factors increased to 68. No adjuvant effect was seen in mice given 10 μg BDP and boosted once. After two boosters, this dose of BDP gave rise to an adjuvant factor of 20 (cf. Table 1).

DOTP gave rise to markedly lower response; no effect was seen after one booster whereas after two booster injections, an adjuvant factor of 4 was seen after administration of 100 μg. TOTM did not show any adjuvant effect in the present bioassay. Neither did MP, which was included as an example of a simple alkyl ester, exhibit an adjuvant effect, which is in agreement with a long-term inhalation study [15].

Data from previously tested substances were modified according to the methods described in the M&M section and the data are included in Table 1.

### IgE and IgG2a responses

No adjuvant effects were seen on IgE, neither after one or two booster injections for any of the studied compounds. However, the BALB/cJ mouse strain tend to respond with high levels of IgE after exposure to OVA and Al(OH)_3_, e.g. [16].

Similarly, the production of IgG2a antibodies was absent or low in all the control groups and test groups, suggesting the absence of a T helper cell type 1 (Th1) response. Only about 12% of the animals responded with a detectable IgG2a level and the mean level of the responders was 79 arbitrary units. However, the mouse strain is capable of responding with high IgG2a levels after OVA exposure. Thus, animals treated with 10 μg OVA and 250 μg of the Th1 adjuvant dimethyldioctadecyl ammonium bromide, cf. [17] and boosted twice with 1 μg OVA in saline, showed a mean IgG2a level of 30,000 arbitrary units (data not shown). Overall, this study demonstrates that the phthalates did not cause isotype switch to production of IgE and IgG2a under the conditions of the test.

## Discussion

Phthalate isomers were studied for their adjuvant effect at different dose levels to obtain information about structure-activity relationships. The different phthalate isomers showed a pronounced variability in enhancing the immunogenicity of OVA. An adjuvant effect was seen as an ability to increase the serum level of OVA-specific IgG1 antibodies, whereas no or limited effects were seen on the production of IgE and IgG2a, fully in line with previous studies [5,7,18].

A number of molecular characteristics play a role for the adjuvant activity. These include:

### Lipophilicity and chain length of the ester groups

For the phthalates, the lipophilicity apparently plays an important role for their adjuvant effect. Lipophilicity increases with the number of carbon atoms in the molecule, which suggests an optimum lipophilicity for adjuvant effect at about the lipophilicity of DEHP (Table 1). This is for example substantiated by the absence of adjuvant activity of TOTM. TOTM contains, like DEHP, two vicinal 2-ethylhexyl esters (cf. Fig 1), but TOTM has, in contrast to DEHP, a third 2-ethylhexyl ester, which increases the lipophilicity. However, the adjuvant effect is not caused by the lipophilicity *per se*, as DOTP, which has the same lipophilicity as DEHP and BDP, has a weak adjuvant effect compared to DEHP and BDP. Furthermore, the non-phthalate substance MP, which has a lipophilicity comparable to DEHP and DBP, had no adjuvant effect. The lack of adjuvant effect of MP is in accordance with a recently published long-term inhalation study [15].

As previously reported, phthalates with 8 carbon atoms in each of the 2 ester groups (a total of 16 carbon atoms) have the maximum adjuvant effect [5]. However, the 16 carbon atoms may be unequally distributed in the ester groups of the phthalate without affecting its adjuvant property. Thus, BDP with chain lengths of 4+12 carbon atoms has an adjuvant effect comparable to that of DEHP with 8+8 carbon atoms. Also, whether the ester chain is branched as in DEHP or linear as in DnOP does not seem to alter the adjuvant effect of the phthalate.

### Distance between the two ester groups

Whether the phthalate is an ortho or a para di-substituted benzene plays a crucial role for the adjuvant effect. Thus adjuvant activity virtually disappears when moving an ester group from the ortho (2') position to the para (4') position as seen from a comparison of DEHP and DOTP (Fig 1 and Table 1).

In conclusion, the adjuvant activity of phthalates depends not only on the lipophilicity but also on the stereochemistry (structure).

## Conclusion

The present study provides information about structure-activity relationship on the adjuvant effect of DEHP-related phthalates and allows a relative comparison of their potencies. Our study shows that even a minor alteration in the structure of a phthalate has dramatic consequences for the adjuvant activity, which allow design of compounds without adjuvant effect but still being useful as plasticizers. Also, as compounds with adjuvant effect caused a strong IgG1 response but no isotype switch to IgE production, it is tempting to speculate whether some of these compounds, after a thorough safety assessment, may have a potential as future vaccine adjuvants.

## Authors' contributions

Both authors were involved in the planning of the study. STL conducted the laboratory work. Both authors contributed to data analyses and preparation of manuscript.

## References

[B1] Nielsen GD, Larsen ST, Olsen O, Lovik M, Poulsen LK, Glue C, Wolkoff P (2007). IgE-mediated sensitisation and airway diseases. Are indoor chemicals adjuvants?. Indoor Air.

[B2] Lycke N (2005). Targeted vaccine adjuvants based on modified cholera toxin. Curr Mol Med.

[B3] Butala JH, David RM, Gans G, McKee RH, Guo TL, Peachee VL (2004). Phthalate treatment does not influence levels of IgE or Th2 cytokines in B6C3F1 mice. Toxicology.

[B4] Larsen ST, Hansen JS, Thygesen P, Begtrup M, Poulsen OM, Nielsen GD (2001). Adjuvant and immuno-suppressive effect of six monophthalates in a subcutaneous injection model with BALB/c mice. Toxicology.

[B5] Larsen ST, Lund RM, Nielsen GD, Thygesen P, Poulsen OM (2002). Adjuvant effect of di-n-butyl-, di-n-octyl-, di-iso-nonyl- and di-iso-decyl phthalate in a subcutaneous injection model using BALB/c mice. Pharmacol Toxicol.

[B6] Hansen JS, Larsen ST, Poulsen LK, Nielsen GD (2007). Adjuvant effects of inhaled mono-2-ethylhexyl phthalate in BALB/cJ mice. Toxicology.

[B7] Larsen ST, Hansen JS, Hansen EW, Clausen PA, Nielsen GD (2007). Airway inflammation and adjuvant effect after repeated airborne exposures to di-(2-ethylhexyl)phthalate and ovalbumin in BALB/c mice. Toxicology.

[B8] Glue C, Platzer MH, Larsen ST, Nielsen GD, Skov PS, Poulsen LK (2005). Phthalates potentiate the response of allergic effector cells. Basic Clin Pharmacol Toxicol.

[B9] Bornehag CG, Sundell J, Weschler CJ, Sigsgaard T, Lundgren B, Hasselgren M, Hagerhed-Engman L (2004). The association between asthma and allergic symptoms in children and phthalates in house dust: a nested case-control study. Environ Health Perspect.

[B10] Larsen ST, Lund RM, Nielsen GD, Thygesen P, Poulsen OM (2001). Di-(2-ethylhexyl) phthalate possesses an adjuvant effect in a subcutaneous injection model with BALB/c mice. Toxicol Lett.

[B11] Larsen ST, Lund RM, Thygesen P, Poulsen OM, Nielsen GD (2003). Investigation of the adjuvant and immuno-suppressive effects of benzyl butyl phthalate, phthalic acid and benzyl alcohol in a murine injection model. Food Chem Toxicol.

[B12] Larsen ST, Nielsen GD, Thygesen P (2002). Investigation of the adjuvant effect of polyethylene glycol (PEG) 400 in BALB/c mice. Int J Pharm.

[B13] Larsen ST, Hansen R, Hammer M, Tegner U, Poulsen OM, Nielsen GD (2004). Adjuvant effect of quaternary ammonium compounds in a murine model. Toxicol Lett.

[B14] Timbrell J (2000). Principles of biochemical toxicology.

[B15] Hansen JS, Larsen ST, Poulsen LK, Nielsen GD (2007). Does lipophilicity per se induce adjuvant effects? Methyl palmitate as model substance does not affect ovalbumin sensitization. J Toxicol Environ Health, Part A.

[B16] Larsen ST, Hansen R, Poulsen OM, Nielsen GD (2004). Adjuvant effect of benzalkonium chloride on the allergen-specific IgE, IgG1 and IgG2a antibody formation in BALB/cJ mice. Basic Clin Pharmacol Toxicol.

[B17] Lindblad EB, Elhay MJ, Silva R, Appelberg R, Andersen P (1997). Adjuvant modulation of immune responses to tuberculosis subunit vaccines. Infect Immun.

[B18] Larsen ST, Nielsen GD (2007). The adjuvant effect of di-(2-ethylhexyl) phthalate is mediated through a PPARα-independent mechanism. Toxicol Lett.

